# Preoperative inspiratory muscle training for patients undergoing cytoreductive surgery and hyperthermic intraperitoneal chemotherapy: a randomized feasibility study

**DOI:** 10.1186/s40814-026-01946-z

**Published:** 2026-10-27

**Authors:** Olivia Sand, Erebouni Arakelian, Egidijus Semenas, Wilhelm Graf, Peter Cashin, Mikael Andersson

**Affiliations:** 1 Department of Surgical Sciences, Gastrointestinal Surgery, Uppsala University, Uppsala, Sweden; 2 Department of Surgical Sciences, Anaesthesiology and Intensive Care, Uppsala University, Uppsala, Sweden; 3 Department of Health Sciences, The Swedish Red Cross University, Stockholm, Sweden; 4 Department of Medical Sciences, Allergy- and Sleep Research, Uppsala University, Respiratory, Uppsala, Sweden; 5 Department of Women’s and Children’s Health, Physiotherapy and Behavioral Medicine Unit, Uppsala University, Uppsala, Sweden

**Keywords:** Inspiratory muscle training, Prehabilitation, Peritoneal metastases, Optimization

## Abstract

**Background:**

Patients undergoing surgery due to peritoneal metastases (PM) are at high risk of postoperative pulmonary complications (PPCs). While several studies have provided promising results regarding the positive effects of preoperative inspiratory muscle training (IMT) regarding length of hospital stay and PPCs after other types of major abdominal surgery, its feasibility in the preoperative management of patients with PM is still unclear. The aim of the study was to evaluate the feasibility of preoperative IMT for patients scheduled for cytoreductive surgery (CRS) and hyperthermic intraperitoneal chemotherapy (HIPEC).

**Methods:**

A single-blinded randomized controlled feasibility study was conducted in Uppsala, Sweden, between 2016 and 2020. Inclusion criteria were age ≥ 18 years, surgery scheduled ≥ 2 weeks after inclusion, and proficiency in Swedish. Patients were randomized 1:1 to either IMT or positive expiratory pressure (PEP). Feasibility was evaluated using two domains: process feasibility (recruitment, intervention adherence, and patient acceptability) and scientific feasibility (ability to perform pre- and postoperative respiratory muscle and lung function assessments, reproducibility of assessments, and adverse events). Preliminary treatment effects and incidence of postoperative pulmonary complications (PPCs) were also evaluated.

**Results:**

Thirty participants were enrolled, with a recruitment rate of 11%. The median training period was 24.5 days, with adherence of 77% in the intervention group and 98% in the control group. Acceptability was high, with 87% rating the training program ≥ 5 on a scale of 1–10. Two participants withdrew due to discomfort at the postoperative assessments, and the reproducibility of assessments was 57–100%. One IMT participant withdrew due to a dry cough. The intervention group showed a larger increase in maximal inspiratory pressure (MIP) from baseline to follow-up and faster recovery of vital capacity (VC) than controls. PPCs were more frequent in the control group.

**Conclusions:**

Preoperative IMT was acceptable to patients and safe, but recruitment and attrition were identified as obstacles to address in a future full-scale RCT.

**Trial registration:**

NCT05665257. Registered 23 December 2022—retrospectively registered.

**Supplementary Information:**

The online version contains supplementary material available at https://doi.org/10.1186/s40814-026-01946-z.

## Key messages regarding feasibility

- Promising results on preoperative IMT have been published, but previous studies have not focused on the aspects related to process feasibility. Furthermore, no studies have investigated the role of preoperative IMT for patients undergoing surgery due to peritoneal metastases. This study was designed to explore two aspects of feasibility before conducting a full-scale RCT.
- Intervention-related aspects of feasibility, such as patient acceptability and the reporting of adverse events, were deemed acceptable. However, challenges related to intervention adherence, recruitment, and attrition were identified.
- The findings of the study led to insights into what aspects would need to improve in a full-scale RCT.

## Introduction

The concept of prehabilitation encompasses nutritional, psychological, behavioral, and physical measures to optimize patients’ health and reduce the risk of complications or impairments related to the disease and its treatment [1]. Among prehabilitation strategies, preoperative inspiratory muscle training (IMT) has shown promising results in improving postoperative outcomes such as reducing length of stay and postoperative pulmonary complications (PPCs) after abdominal and thoracic surgery [2–4].

Cytoreductive surgery (CRS) with intraperitoneal chemotherapy (HIPEC) has become the standard treatment for peritoneal metastases (PM) [5], and although this procedure has markedly improved survival, it remains associated with a high risk of PPCs, one of the most common complications following CRS and HIPEC [6–9].

Given the promising results of IMT before other abdominal and thoracic procedures [2, 3], we aimed to assess the feasibility of IMT before CRS and HIPEC. A potential challenge in this setting is the short interval between diagnosis and surgery. According to Swedish national colorectal cancer guidelines, long-term survival depends on minimizing time to surgery [10], leaving only a few weeks, or less, for prehabilitation. Since measurable effects seem to require at least 2 weeks of IMT [4], this limited window may hinder implementation. The Enhanced Recovery after Surgery Society also recommends routine prehabilitation for patients scheduled for CRS and HIPEC [11]. Despite the established effects of IMT on length of stay and PPCs [2–4], it is not widely implemented in Swedish practice. Moreover, most previous IMT studies have applied relatively low training loads [12–15], although effective muscle strengthening requires higher intensity [16].

To address these issues, the study aimed to evaluate two aspects of feasibility of preoperative IMT in patients with PM before conducting a larger randomized controlled trial.

## Materials and methods

### Participants and study designs

A single-blinded randomized controlled feasibility study was performed at Uppsala University Hospital, Sweden, between 2016 and 2020. Consecutive sampling was applied to enhance recruitment. Adult (age ≥ 18) patients scheduled for CRS and HIPEC were eligible for inclusion, whereas those scheduled for surgery < 14 days from inclusion and/or unable to understand written and oral Swedish were ineligible. Participants who were identified as inoperable at the time of the surgery due to an advanced tumor burden, and hence, did not receive HIPEC, were excluded postoperatively from the study. However, preoperative data from these participants were used in the analyses. The chosen sample size, *n* = 30, was based on the estimated number of patients undergoing CRS and HIPEC each year, 60–70 patients, and an expected inclusion rate of ≥ 50%. This was chosen based on feasibility of inclusion, 50% as the predefined progression criterion, and diminishing benefits in precision when including ≥ 30 participants [17]. Ethical permission was obtained from the Uppsala Swedish Ethical Review Authority (reference no. 2016/338).

### Randomization and blinding

Participants were randomized 1:1 to the intervention or control group using sealed envelopes prepared by an independent physiotherapist. Baseline assessments were completed before allocation. Participants were informed that two preoperative breathing exercise programs were compared, but were unaware which was the experimental condition, thereby maintaining single blinding.

### Procedure

Recruitment took place at a scheduled appointment at the outpatient clinic 2–10 weeks before surgery. The physiotherapist in the study provided patients with oral and written information about the study, and written consent was obtained from those agreeing to participate. Baseline assessments (*t*_−1_) included inspiratory vital capacity (VC), forced expiratory volume during the first second (FEV_1_), and maximal inspiratory pressure (MIP) (Table 1). Demographic data, such as age, body mass index (BMI), Karnofsky performance status, and comorbidity, were also collected. A follow-up assessment (*t*_1_) of MIP 1 to 4 days before surgery was performed to evaluate potential intervention effects. We also collected data on physical activity level, fatigue, and patient satisfaction and acceptability using questionnaires from the Swedish National Board of Health and Welfare about physical activity (NBHW-PA) [18, 19], the Swedish-translated version of the Multidimensional Fatigue Inventory (MFI-20) [20], and a study-specific questionnaire inspired by Hulzebos et al. [21] (Appendix 1). Examples of questions in the questionnaire were “What was your overall experience of the training program?”, “How would you rate the training program from 1 to 10?” and “Would you consider participating in a study like this again?”. The two postoperative assessments on days 2 and 7 (*t*_2_ and *t*_3_, respectively) after surgery included VC and MIP.

**Table 1:** Schedule of enrollment, interventions, and assessments

		Enrollment	Allocation	Post-allocation			Close-out
Timepoint*		*−t*_*1*_	*t*_*0*_	*t*_*1*_	*t*_*2*_	*t*_*3*_	*t*_*4*_
Enrollment	Eligibility screen	x					
	Informed consent	x					
	Allocation		x				
Interventions	Intervention: IMT		x	x			
	Control: PEP		x	x			
Assessments	Baseline variables:						
Anthropometrics	Body height, body weight	x					
History of smoking	Interview	x					
Karnofsky performance status	Medical records	x					
Comorbidity	Medical records	x					
Physical activity level	NBHW-PA			x			
Fatigue	MFI-20			x			
	Process feasibility:						
Recruitment							x
Time available for training							x
Intervention adherence	Diary						x
Patient acceptability	Study-specific questionnaire			x			
	Scientific feasibility:						
Ability to perform VC, FEV1, and MIP							x
Reproducibility of tests							x
Lung function tests	Inspiratory VC	x			x	x	
	FEV_1_	x					
Respiratory muscle strength test	MIP	x		x	x	x	
Postoperative pulmonary complications	Medical records						x
Comprehensive Complication Index	Medical records						x
Adverse events	Diary			x			

^*^t_*−1*_ = enrollment and baseline assessments*, t*_*0*_ = randomization*, t*_*1*_ = 1 to 4 days before surgery, *t*_*2*_ = postoperative day *2*,* t*_*3*_ = postoperative day 7, *t*_*4*_ = close-out, after discharge from the hospital

*IMT* inspiratory muscle training, *PEP* positive expiratory pressure, *NBHW-PA* National Board of Health and Welfare Physical Activity Questionnaire, *MFI-20* Multidimensional Fatigue Inventory, VC vital capacity, *FEV*_*1*_ Forced expiratory volume during the first second,* MIP* maximal inspiratory pressure

For the assessments of inspiratory VC and FEV_1_, we used a handheld spirometer (SpiroPerfect™ PC-Based Spirometer, Welch Allyn, New York, USA). MIP was assessed using an electronic handheld device (PowerBreathe KH2, HaB International Ltd., Southam, England) connected to a software (Breathe-Link Medic Live feedback Software, HaB International Ltd., Southam, England) for the collection and analysis of the data. Lung function and inspiratory muscle strength were assessed according to international standards, and reproducibility was defined as < 150 ml difference for VC and 10% difference for MIP [22–26].

Data regarding PPCs were retrieved from medical records and were graded according to the Clavien Dindo classification of surgical complications [27]. To further describe the incidence of PPCs, we also used the comprehensive complication index (CCI), which integrates all recorded complications with their respective severity [28]. While the Clavien-Dindo scale considers only the most severe complication, the CCI incorporates both the number and severity of complications, yielding a score between 0 and 100. All PPCs that occurred during the hospital stay following surgery were recorded.

## Intervention

### Intervention group

Participants in the intervention group were assigned to IMT using a handheld electronic device from PowerBreathe (Powerbreathe K3, HaB International Ltd., Southam, England), which they were instructed to use twice daily for at least 2 weeks. Each session consisted of 30 × 2 breaths, facilitated by the countdown feature in the K3, which automatically counts down from the preset 30 breaths. Resistance was set to 50% of the baseline MIP, and the electronically controlled valve of the device ensured that the relative resistance was maintained throughout the entire inspiratory phase. Nose clips were used during each session. The Borg rating of perceived exertion scale [29] was used to rate the intensity. Participants were instructed to increase the resistance by 7 cmH2O each time they rated the intensity below 15. To assess compliance, participants were given a diary and asked to record their training details. Adverse events, defined as any event causing the participant discomfort, pain, or other disturbing sensations, were also to be registered in this diary.

### Control group

Participants in the control group were assigned to a program that used a positive expiratory pressure (PEP) device (Mini-PEP, Dolema AB, Sollentuna, Sweden), which was considered a sham treatment. Participants were instructed to use the PEP device twice a day, 3 × 10 breaths each session, for at least 2 weeks. Moreover, they were also given a diary and were asked to provide the same type of information as the intervention group.

### Outcome measures

To describe the different aspects of feasibility, we used the classification system presented by Thabane et al. [30] and focused on two domains: process feasibility and scientific feasibility. Process feasibility was evaluated based on recruitment, available training time, intervention adherence, and patient acceptability. Scientific feasibility was evaluated by the ability to perform pre- and postoperative respiratory muscle strength and lung function tests, the reproducibility of these tests, and the presence of adverse events. In addition to feasibility outcomes, we evaluated preliminary treatment effects and variance, the incidence of PPCs, and the median comprehensive complication index in the two groups. However, these were not considered feasibility outcomes.

### Process feasibility

Recruitment rate was defined as the number of included participants divided by the number of potentially eligible patients between November 2016 and September 2020. Time available for training was calculated as the number of days from inclusion to the day before the MIP follow-up assessment (*t*_1_). Intervention adherence was calculated as the number of sessions each participant recorded in their diary divided by the number of potential sessions before surgery. Patient acceptability was assessed by the study-specific questionnaire on experiences of the study and training program, and feasibility was evaluated based on question number seven, “How would you rate the training program from 1 to 10?”.

Progression criteria related to process feasibility.

- Recruitment ≥ 50%
- Time available for training ≥ 14 days
- Adherence rate ≥ 80%
- Patient acceptability was deemed good if ≥ 75% of those who answered rated the training program 5 or higher

#### Scientific feasibility

To evaluate scientific feasibility, we analyzed the ability to perform pre- and postoperative respiratory muscle and lung function assessments and the proportion of assessments that were reproducible according to official guidelines [23]. Information about adverse events was retrieved from participants’ diaries.

Progression criteria related to scientific feasibility:

- The proportion of participants, still included in the study after surgery, who performed all respiratory muscle and lung function assessments ≥ 75%
- The proportion of participants who performed reproducible respiratory muscle and lung function assessments ≥ 75% at all tests
- No reporting of adverse events defined as moderate or worse according to the Common Terminology Criteria for Adverse Events version 5.0 [31]

Furthermore, treatment effects were assessed by evaluating within-group differences in MIP and VC, the incidence of PPCs in each group using the Clavien–Dindo classification for surgical complications [27] and calculating the total CCI [28] in each group.

### Statistical analysis

All data analyses were performed using IBM SPSS Statistics for Windows version 23 (IBM Corp, Armonk, NY, USA). Demographic continuous data were presented as median and interquartile range (IQR), whereas categorical data were presented as frequencies and percentages. Continuous feasibility data were presented using median, interquartile range (IQR), mean, standard deviation (SD), and 95% confidence intervals (CI). Categorical feasibility data were presented using frequencies and percentages. All analyses on preliminary treatment effects were conducted on all available data at each respective time point. No formal hypothesis testing was performed due to the nature of feasibility studies.

## Results

**Fig. 1:**
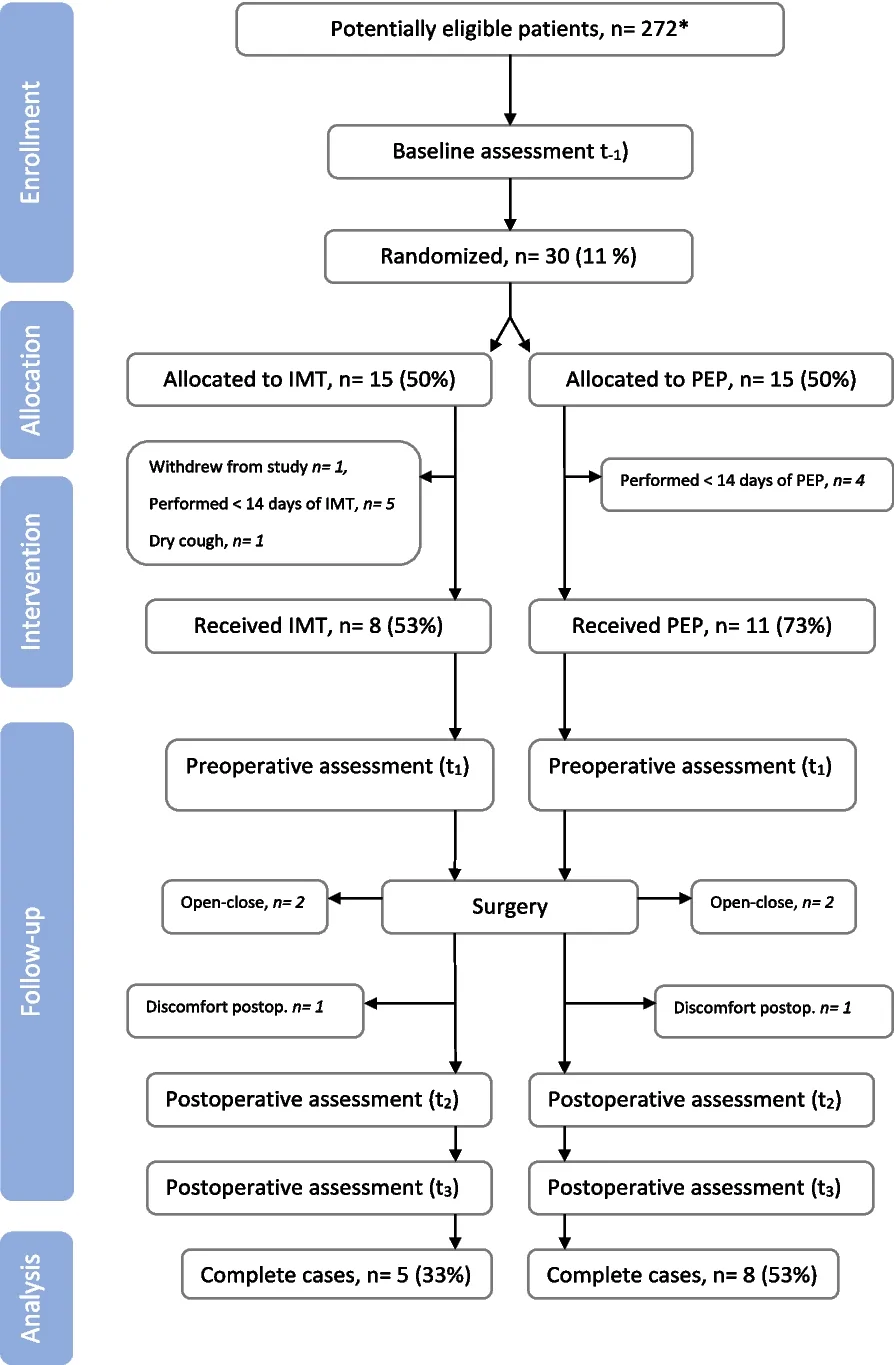
Flow chart over inclusion and attrition. *We do not have data on who was ineligible or who declined participation. *t*_*−1*_ = enrollment and baseline assessments *t*_*0*_ = randomization *t*_*1*_ = 1 to 4 days before surgery *t*_*2*_ = postoperative day 2 *t*_*3*_ = postoperative day 7 *IMT* Inspiratory muscle training *PEP* Positive expiratory pressure

A total of 30 participants were included in the study; 17 chose to withdraw or were excluded (Fig. 1). One participant in the intervention group found the program too demanding and withdrew from the study immediately after randomization. This participant, however, had already signed the consent form and was formally included. We included all available baseline data for this individual in Tables 2 and 4, but all further analyses were conducted with a maximum of 29 participants.

**Table 2:** Demographic data of the included participants

	IMT (*n* = 15)	PEP (*n* = 15)
Gender, *n* (%)		
Female	11 (73.3)	8 (53.3)
Age, median (IQR)	66 (56–70)	65 (56–73)
BMI	26.4 (22.3–32.6)	26.0 (24.0–29.9)
Smoking^a^	5 (33.3)	7 (46.6)
FEV_1_	2.5 (2.0–2.8)	2.4 (1.9–3.0)
Karnofsky performance status	100 (90–100)	100 (80–100)
Comorbidity (yes)	9 (60.0)	9 (60.0)
COPD	0 (0)	1 (7)
Exercise (min/week)*		
0 min	5 (35.7)	6 (40.0)
< 30	2 (14.3)	2 (13.3)
30–60	3 (21.4)	3 (20.0)
90–120	1 (7.1)	1 (6.7)
> 120	1 (7.1)	1 (6.7)
Missing	2 (14.3)	2 (13.3)
Physical activity (min/week)*		
90–150	4 (28.6)	3 (20.0)
150–300	3 (21.4)	5 (33.3)
> 300	4 (28.6)	5 (33.3)
Missing	3 (21.4)	2 (13.3)
Sedentary time (min/day)*^b^		
60–180	3 (21.4)	3 (20.0)
240–360	6 (42.9)	7 (46.7)
420–540	3 (21.4)	2 (13.3)
780–900		1 (6.7)
Missing	2 (14.3)	2 (13.3)
MFI-20 (total score)*	42 (30.0–50.3)	46 (34.0–57.5)

*IMT* inspiratory muscle training, *PEP* Positive expiratory pressure, *IQR* interquartile range, *BMI* body mass index, *FEV*_*1*_ forced expiratory volume during the first second, *MFI-20* Multidimensional Fatigue Inventory, *COPD* chronic obstructive pulmonary disease, *Exercise* Refers to vigorous intensity physical exercise *Physical activity* Refers to moderate intensity physical activity

^*^These variables contain missing data from the participant who withdrew from the study after randomization

^a^Ever smoker

^b^Sleep excluded

### Process feasibility

During the recruitment period, November 2016–September 2020, 272 patients underwent CRS and HIPEC at Uppsala University Hospital, corresponding to a recruitment rate of 11% (Fig. 1).

Of the 29 participants who received the two questionnaires at baseline, 24 completed the questionnaire on patient acceptability (Table 3). We chose to present data from all participants who completed the questionnaire, including those who had performed < 14 days of IMT/PEP. As shown in Table 3, some data are missing. The reasons for this are withdrawal from the study during the intervention period, surgery too close to inclusion and therefore exclusion, and forgetting to return the questionnaire. The patient who forgot to return the questionnaire was later excluded from the study because the individual was considered inoperable. When asked to rate the training program on a scale of 1 to 10, 9/11 (82%) of participants in the IMT group and 13/13 (100%) in the PEP group rated it 5 or higher. In the IMT group, 7/9 (78%) marked yes, when asked whether they would consider participating in a similar research study again. Those numbers were 12/13 (92%) in the PEP group.

**Table 3:** Patient acceptability for the given intervention

Question:	IMT (11)	PEP (13)
1. What was your overall experience of the training program?		
- Not very good	1	
- Good	6	10
- Very good	4	3
2. How would you rate the intensity of the training?		
- Easy		6
- Moderate	10	6
- Strenuous	1	1
3. How would you describe the length of the training period?^*^		
- Good	9	9
- Too long	2	2
- Short		1
4. What was your level of motivation?		
- Thought about quitting	3	
- Motivated	2	9
- Very motivated	6	4
5. Did you experience any training effects?^*^		
- Yes	5	5
- No		1
- Unsure	6	6
6. Would you consider participating in a study like this again?		
- Yes	7	12
- No	2	1
7. How would you rate the training program 1–10?^*^		
- < 5	1	
- 5	1	1
- > 5	8	12
8. How would you rate your motivation during the training period 1–10?^*^		
- < 5	2	
- 5	1	
- > 5	7	13

*IMT* inspiratory muscle training, *PEP* positive expiratory pressure

^*^There were missing data in four of the questions: 3, 5, 6, 7, and 8

The overall median time available for training was 24.5 days (IQR 16–33). The median time available for training was 26.5 (IQR 16–46) days in the intervention group and 23 (IQR 16–29) days in the control group. The median adherence rate was 77% (IQR 45–100) in the intervention group and 98% (IQR 95–100) in the control group. The median Borg rating across all training sessions was 15 (IQR 13–16).

### Scientific feasibility

The overall percentage of patients performing reproducible MIP, VC, and FEV_1_ maneuvers ranged from 57 to 100% (Table 4).

**Table 4:** Reproducibility rates from the pre-and postoperative. assessments of maximal inspiratory muscle strength and lung function

Reproducible assessment attained								
	Baseline MIP (*t*_−1_)	Baseline VC (*t*_−1_)	Baseline FEV_1_ (*t*_−1_)	Follow-up MIP (*t*_1_)	Postop day 2 MIP (*t*_2_)*	Postop day 2 VC (*t*_2_)*	Postop day 7 MIP (*t*_3_)*	Postop day 7 VC (*t*_3_)*
IMT	13/15 (87%)	13/15 (87%)	15/15 (100%)	8/8 (100%)	4/4 (100%)	4/4 (100%)	4/5 (80%)	4/5 (80%)
PEP	13/15 (87%)	14/15 (93%)	14/15 (93%)	9/11 (82%)	6/7 (86%)	4/7 (57%)	8/8 (100%)	7/8 (88%)

*IMT* inspiratory muscle training, *PEP* positive expiratory pressure, *MIP* maximal inspiratory pressure, *VC* vital capacity, *FEV*_*1*_ forced expiratory volume during the first second

^*^One participant in each group was physically unfit to perform the t_2_ assessments but performed the *t*_3_ assessments

Two participants in each group registered adverse events in their diaries. One participant in the intervention group experienced a dry cough, nausea, and headache, and chose to withdraw from the study due to the inconvenience caused by the dry cough. The second participant in the intervention group who reported an adverse event also experienced a dry cough but continued participating in the study. In the control group, one participant reported shortness of breath, and one reported retching. Neither of these participants ended their participation.

**Fig. 2:**
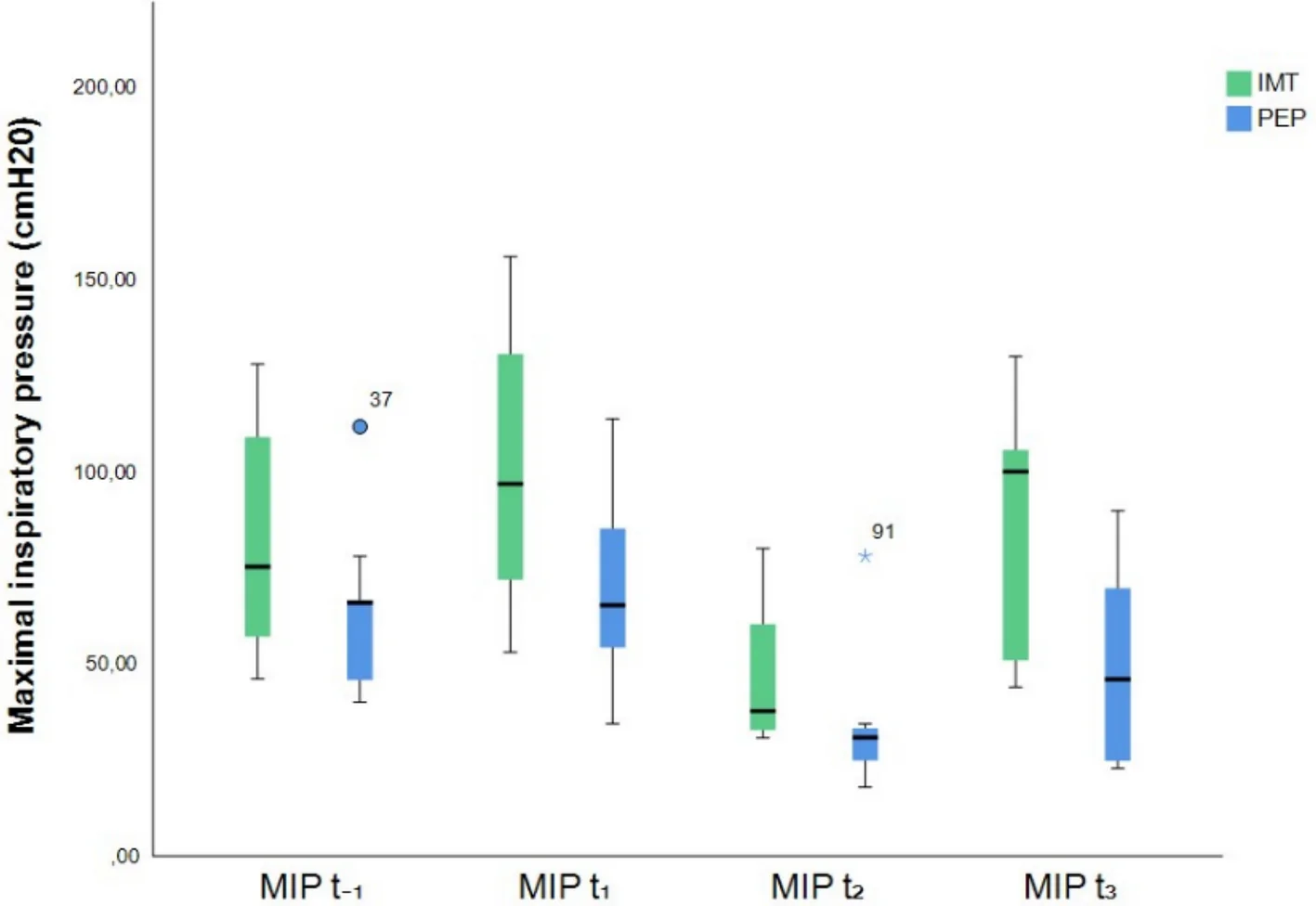
Box plot on all MIP assessments*. MIP* Maximal inspiratory pressure *t*_*−1*_ Baseline assessment *t*_*1*_ Preoperative follow-up assessment *t*_*2*_ Postoperative assessment day *2 t*_*3*_ Postoperative assessment day 7 *IMT* Inspiratory muscle training *PEP* Positive expiratory pressure

**Fig. 3:**
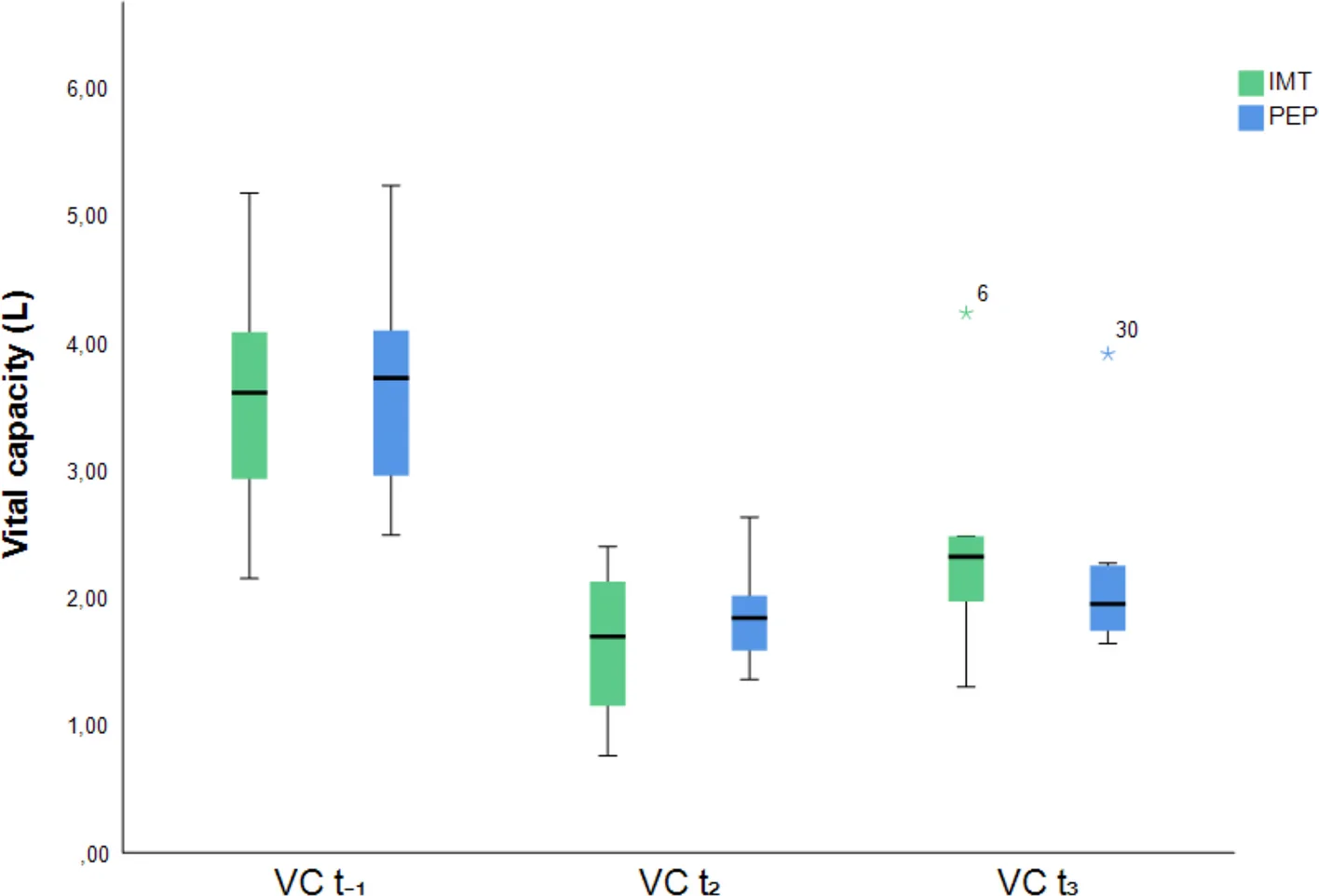
Box plot on all VC assessments*. VC* Vital capacity *t*_*−1*_ Baseline assessment *t*_*2*_ Postoperative assessment day 2 *t*_*3*_ Postoperative assessment day 7 *IMT* Inspiratory muscle training *PEP* Positive expiratory pressure

The intervention group had a larger increase in MIP between baseline (*t*_−1_) and follow-up (*t*_1_) (median 15 cmH2O, IQR 12.7–27.4, mean 18 cmH2O, SD 8, 95% CI 11.9–24.2) compared to the control group (median 2 cmH2O, IQR − 7.1–16.5, mean 6 cmH2O, SD 12.8, 95% CI − 2.6–14.6 (Fig. 2). The intervention group also had a larger recovery in VC between the first (*t*_2_) and second (*t*_3_) postoperative assessment (median 0.5 L, IQR 0.4–1.5, mean 0.8 L, SD 0.7, 95% CI − 0.3–1.9) compared to the control group (median 0.1 L, IQR − 0.1–0.3, mean 0.1 L, SD 0.2, 95% CI − 0.2–0.3) (Fig. 3).

The incidence of PPCs was higher in the control group (n = 12) compared to the intervention group (*n* = 6). Out of the 12 PPCs in the control group, one was a pulmonary embolism, three were pleural effusion, one was pneumonia, and one was a respiratory failure that required non-invasive ventilation. In the IMT group, two patients had pleural effusion. Hypoxia and atelectasis were prevalent in both groups. The median CCI was 8.7 (IQR 6.5–16.4, mean 9.7, SD 9.6, 95% CI 0.9–18.6) in the intervention group and 8.7 (IQR 8.7–16.1, mean 14.6, SD 15.4, 95% CI 3.6–25.6) in the control group.

## Discussion

This randomized feasibility trial demonstrated that a future IMT trial is feasible to conduct if certain aspects regarding recruitment and attrition are considered. Considering the potential clinical benefits in PPCs, a future full-scale trial is warranted.

In previous research from our institution, we have investigated the incidence of PPCs after CRS and HIPEC [9]. Interestingly, PPCs were determined to be an independent risk factor for worse overall survival. Considering the potential clinical benefit identified in this feasibility study, improving the rate of PPCs after CRS and HIPEC may, in turn, improve overall survival. It is relevant to explore this possibility of not just improving PPCs, but overall survival as well.

Previous IMT trials before abdominal surgery have reported recruitment challenges [32, 33], and our rate may appear especially low. However, we estimated recruitment using all 272 operated patients as the denominator because eligibility at the time was not fully captured. Hence, the true rate among eligible patients was likely higher. We used consecutive sampling and extended the recruitment period, but several contextual factors limited accrual. One study year had very short surgical wait times (< 2 weeks), leaving insufficient time for study inclusion and training. This is particularly important since, although there is currently no consensus regarding the optimal duration of preoperative IMT, previous systematic reviews suggest that preoperative IMT should ideally be performed for at least 2 weeks [3]. Notably, registry data from Uppsala (2016–2019) show a mean of 52 days from decision to surgery, indicating a workable window for prehabilitation when processes are aligned. A practical improvement is to shorten the interval between listing for surgery and the first preoperative clinic visit, allowing prehabilitation to start earlier.

Recruitment was also affected by operational factors, as having only one physiotherapist dedicated to enrolment led to some eligible patients being missed. Patients are often approached for multiple studies, and coordinating study information/consent could reduce burden and improve uptake. Furthermore, timing matters, and inviting patients immediately after the surgical consultation, when stress is high and schedules are tight, limits opportunities to discuss participation. Future trials should engage regional hospitals to begin outreach soon after diagnosis, allocate additional staffing for screening and consent, and schedule information sessions separate from surgical visits. These pragmatic adjustments should enhance recruitment, reduce selection risk, and improve external validity.

Another challenge was the high attrition. The major cause of attrition was less than 14 days of prehabilitation time. As mentioned earlier, logistical planning may alleviate this issue. Next, the rate of open-close surgery also led to exclusion. Despite the use of CT scans and diagnostic laparoscopy, the true tumor burden is often unknown until the date of the CRS and HIPEC. In our study, almost 17% were identified as inoperable at the time of surgery. These numbers correspond to previous research reporting that approximately 20% of the patients scheduled for CRS and HIPEC are considered inoperable [34, 35], which naturally results in higher attrition than at other abdominal cancer surgeries. On the other hand, patients undergoing full explorative laparotomy could also be included in future research. Even though the IMT treatment may have less impact, it will presumably improve postoperative recovery in this patient group as well.

The challenge of postoperative lung assessments is well known and has been reported previously in patients undergoing cardiac and abdominal surgery [36, 37]. Scheduling the first assessment 2 days after surgery was likely too early; had it been on day 3 or 4, more participants might have been fit to continue. Although day 2 was chosen to capture the expected immediate postoperative decline and recovery in VC and MIP [15, 38], the high attrition rate suggests limited value, as these early measurements were not essential to the study’s primary aim.

Self-reported lifestyle data should always be treated with caution due to the widely recognized risk of overestimation [39, 40]. In our study, adherence to the intervention was marginally below the preset limit in the intervention group (77% vs 80%), but acceptable in the control group. It has been suggested that factors such as supervision, initial exploration of participants’ characteristics, facilitators and barriers, goal setting, and communication and feedback improve adherence to physical exercise [41]. These are potential improvements to consider in future intervention studies.

Physical exercise during cancer treatment has beneficial effects on perceived physical and psychological well-being and promotes the feeling of control and meaningfulness in an otherwise unpredictable situation [42, 43]. These are important aspects of prehabilitation that go beyond the classic focus on postoperative medical outcomes and support the value of prehabilitation in cancer care. A majority of patients who completed the study-specific questionnaire reported a positive experience with the program, and most ranked their motivation as high. This is an important indicator that a larger RCT would be feasible from a patient acceptability perspective, and it aligns with previous evaluations of preoperative IMT programs before abdominal surgery [32].

Adverse events were reported by two participants in each group, which had not been reported as an issue in previous IMT studies [3]. In the intervention group, two participants reported a dry cough; one continued, while one withdrew. Our liberal definition of adverse events, any event causing discomfort, pain, or unexpected sensations, may explain this difference, as earlier studies often left the term undefined [21, 44]. Participants were also not informed about possible harmless sensations, which may have lowered tolerance. Furthermore, the higher training load in this study could also have contributed. Overall, the number and severity of events were considered acceptable. A summary of all progression criteria and considerations for future trials is presented in Table 5.

**Table 5:** Considerations for future trials

Aspects of feasibility	Progression criteria	Challenges and considerations
Process feasibility		
Recruitment rate	≥ 50%	The recruitment rate was low mainly due to: - Insufficient staffing dedicated to recruiting - Short wait times - Timing of recruitment and patients not having time to receive information about the study These challenges may be solved by adding more resources, not only locally but also by involving more centers, to enable a more effective recruitment and optimizing the wait times between diagnosis and surgery. Informing patients before their first visit at the hospital that they would be asked to participate in a study might have also improved the number of patients able to participate
Time available for training	≥ 14 days	Nine participants were excluded due to insufficient training even though data support that there should be enough time between diagnosis and surgery to allow for 2 weeks of training. Scheduling patients for their first outpatient appointment earlier or involving more centers could be two ways of ensuring a period long enough to have sufficient time for training
Intervention adherence	≥ 80%	Intervention adherence was above 80% in the control group but just below the limit in the intervention group. Supervised training, exploration of participants’ characteristics, facilitators and barriers, goal setting, and feedback may all be interesting additions to a future study protocol to improve intervention adherence
Patient acceptability	≥ 75% of those who answered rated the training program 5 or higher	Patient acceptability was deemed feasible in both groups
Scientific feasibility		
Ability to perform pre- and postoperative lung function and respiratory muscle strength tests	≥ 75% of those still included in the study postoperatively	Less than 75% of those still included in the study after surgery where able to perform the postoperative tests. Our suggestion would be to remove the postoperative tests from the study protocol in a future trial if not central to the objectives of the study
Reproducibility of tests	≥ 75% at all tests	The postoperative tests were proven challenging even to those able to perform them and reproducibility of tests were > 75% in all tests except the first postoperative test in which only 57% of the control group attained reproducible results. This is another reason to consider not including them in future trials
Adverse events	No reporting of adverse events defined as moderate or worse according to the Common Criteria for Adverse Events version 5.0	There were no issues regarding adverse events, only mild adverse events were reported

Although treatment effects were not the primary focus, we included exploratory data since this is, to our knowledge, the first study evaluating preoperative IMT in patients with PM. The intervention group showed a greater increase in MIP from baseline to follow-up and faster postoperative recovery of VC, consistent with previous findings [12, 37]. These observations are consistent with the expected physiological effects of IMT. By improving inspiratory muscle strength, IMT is expected to increase MIP, which may facilitate deep breathing postoperatively and thereby promote a faster recovery of VC [3, 45]. As postoperative care was standardized across groups, the observed differences are unlikely to be explained by differences in postoperative management. However, as this feasibility study was not powered to evaluate treatment efficacy, the exact contribution of IMT to these improvements cannot be established.

In Uppsala, out-of-region patients are typically discharged 7–10 days after surgery, limiting data collection to the early postoperative period. Thus, 30- or 90-day morbidity could not be assessed, likely underestimating the true incidence of PPCs and affecting CCI results. The most recent meta-analysis on preoperative IMT before abdominal surgery reported a 50% reduction in PPC risk among trained patients [4]. Our study was not designed for hypothesis testing; hence, our results are not directly comparable.

A key strength of this study was that pre- and postoperative management remained unchanged, allowing a realistic assessment of feasibility for a future RCT. This approach helped identify several protocol- and intervention-related challenges to address before scaling up. High attrition led to missing outcome data, reducing internal validity, but also revealed important pitfalls, including reliance on a single physiotherapist for enrolment and assessments, the burden of postoperative MIP and VC testing, and insufficient patient information about potential IMT-related discomforts.

## Conclusion

We found that preoperative IMT before CRS and HIPEC was feasible in terms of patient acceptability and safety, and that there appears to be a clinically relevant benefit. Protocol-related issues, such as ineffective recruitment and high attrition rates, as well as intervention-related issues, such as adherence, have provided valuable feedback to plan a future full-scale RCT.

## Supplementary Information

Supplementary Material 1: Appendix 1. Survey on the experience of the training program

## Data Availability

The data underlying the results presented in this study contain potentially sensitive and identifying participant information and cannot be shared publicly due to GDPR. Data are available upon request from the Uppsala University registrar (registrator@uu.se; reference KIRVET 2023/5).

## Abbreviations

CRS: Cytoreductive surgery
HIPEC: Hyperthermic intraperitoneal chemotherapy
IMT: Inspiratory muscle training
MIP: Maximal inspiratory pressure
RCT: Randomized controlled trial
PEP: Positive expiratory pressure
PM: Peritoneal metastases
PPCs: Postoperative pulmonary complications
VC: Vital capacity
