# Supplementary material for: Preoperative inspiratory muscle training for patients undergoing cytoreductive surgery and hyperthermic intraperitoneal chemotherapy: a randomized feasibility study
Source: Pilot Feasibility Stud. 2026 Oct 27;12:152. doi: 10.1186/s40814-026-01946-z (PMC13711825; doi:10.1186/s40814-026-01946-z)
Supplement: Supplementary file 1 [file 40814_2026_1946_MOESM1_ESM.docx]

Appendix 1. Survey on the experience of the training program

1. What was your overall experience of the training program?

Not very good, Good, Very good

2. How did you perceive the intensity?

Easy, Moderate, Heavy

3. What did you think of the length of the training period?

Short, Just right, Too long

4. How motivated were you?

Though about quitting, Motivated, Highly motivated

5. Did you experience any training effects?

Yes, Unsure, No

6. Would you consider participating in a study like this again?

Yes, No

7. How would you rate the training program from 1–10? 1=very bad, 10=very good

1 2 3 4 5 6 7 8 9 10

8. How would you rate your motivation 1–10? 1=very low, 10=very high

1 2 3 4 5 6 7 8 9 1
